# Congenital Rubella Syndrome in the Post-Elimination Era: Why Vigilance Remains Essential

**DOI:** 10.3390/jcm14113986

**Published:** 2025-06-05

**Authors:** Livian Cássia De Melo, Marina Macruz Rugna, Talita Almeida Durães, Stefany Silva Pereira, Gustavo Yano Callado, Pedro Pires, Evelyn Traina, Edward Araujo Júnior, Roberta Granese

**Affiliations:** 1 Discipline of Woman Health, Municipal University of São Caetano do Sul (USCS), São Caetano do Sul 09521-160, São Paulo, Brazil; livian.melo@uscsonline.com.br (L.C.D.M.); marina.rugna@uscsonline.com.br (M.M.R.); talita.duraes@uscsonline.com.br (T.A.D.); stefany.pereira@uscsonline.com.br (S.S.P.); araujojred@terra.com.br (E.A.J.); 2 Albert Einstein Israelite College of Health Sciences, Albert Einstein Israelite Hospital, São Paulo 05653-120, São Paulo, Brazil; gycallado@gmail.com; 3 Department of Obstetrics and Gynecology, University of Pernambuco (UPE), Recife 50100-010, Pernambuco, Brazil; pedropiresfn@gmail.com; 4 Department of Obstetrics, Paulista School of Medicine—Federal University of São Paulo (EPM-UNIFESP), São Paulo 04023-062, São Paulo, Brazil; etraina@unifesp.br; 5 Department of Biomedical and Dental Sciences and Morphofunctional Imaging, “G. Martino” University Hospital, 98100 Messina, Italy

**Keywords:** rubella, pregnancy, immunization, intrauterine infection, congenital rubella syndrome, fetal malformations

## Abstract

Congenital Rubella Syndrome (CRS) results from maternal infection with the rubella virus during pregnancy, particularly in the first trimester, when the risk of vertical transmission and severe fetal damage is highest. CRS is characterized by a broad spectrum of congenital anomalies, including sensorineural hearing loss, congenital heart defects, cataracts, neurodevelopmental delay, and behavioral disorders. Despite the absence of specific antiviral therapies, active immunization remains the only effective strategy to prevent rubella infection and its congenital consequences. Global immunization efforts, particularly in the Americas, have led to the elimination of rubella and CRS in several countries. However, challenges persist in the post-elimination era, including declining vaccine coverage, vaccine hesitancy, and setbacks caused by the COVID-19 pandemic. Diagnosis relies on maternal serology, fetal imaging, postnatal antibody testing, and molecular techniques. Management requires long-term, multidisciplinary follow-up due to the complex and lifelong sequelae affecting sensory, motor, and cognitive development. This review highlights the clinical, epidemiological, and pathophysiological aspects of CRS, while emphasizing the urgent need to maintain high vaccination coverage and strengthen surveillance systems. Sustained public health commitment is essential to prevent the reemergence of rubella and protect future generations from this preventable syndrome.

## 1. Introduction

Rubella is a contagious viral infection that, although typically benign in children, poses a significant risk to pregnant women and their fetuses due to the virus’s high teratogenic potential [1]. When maternal infection occurs during pregnancy, vertical transmission to the fetus may ensue, leading to congenital infection and the development of Congenital Rubella Syndrome (CRS). This intrauterine infection can result in a wide spectrum of malformations and health complications in the newborn and may result in miscarriage or stillbirth [2].

CRS is characterized by a myriad of fetal anomalies, including cataracts, sensorineural hearing loss, congenital heart defects, cerebral damage such as cerebral palsy, and impairments in neurodevelopment [1,2,3,4]. Furthermore, CRS is classified among the STORCH infections (Syphilis, Toxoplasmosis, Other infections, Rubella, Cytomegalovirus, and Herpes simplex), which are known to cause congenital syndromes, adverse neurological outcomes, and, in some cases, permanent disabilities in children [2].

Given the significant impact of rubella and CRS and the absence of specific antiviral therapies, active immunization remains the only effective preventive strategy against rubella virus infection and its associated congenital syndrome. CRS represents the leading cause of vaccine-preventable congenital anomalies [5,6].

Vaccination is widely recognized as one of the safest and most cost-effective public health interventions, playing a central role in reducing the morbidity and mortality associated with vaccine-preventable diseases [7]. In this context, organizations such as the World Health Organization (WHO) and the Pan American Health Organization (PAHO) have played a critical role in promoting and expanding immunization coverage, especially through the Expanded Program on Immunization (EPI) [5]. Structured around the principles of primary healthcare, this program has been essential in the control, elimination, and even eradication of vaccine-preventable diseases. The rubella vaccine is strongly recommended by the WHO, which advocates for its universal inclusion in national immunization schedules, preferably leveraging existing measles control infrastructure [1,3,6].

The elimination goal for measles and rubella in the Americas was set in 1994, leading to a series of coordinated strategies, including maintaining high vaccine coverage, integrating epidemiological surveillance systems, and conducting mass vaccination campaigns [5]. From 2003 onwards, targeted campaigns for adolescents and adults were implemented to accelerate rubella elimination, while follow-up vaccination campaigns in children helped maintain measles control. Integrated surveillance for measles and rubella was pivotal to this process, utilizing combined immunobiological agents, such as the measles–rubella (MR) vaccine or the measles–mumps–rubella (MMR) vaccine [7]. These efforts culminated in the Americas becoming the first WHO region declared free of rubella and Congenital Rubella Syndrome in April 2015, and free of measles in September 2016 [5].

However, sustaining these achievements remains challenging amid the resurgence of vaccine-preventable diseases in several parts of the world. Contributing factors include ongoing viral circulation, low vaccine coverage in low- and middle-income countries, and the negative impact of the COVID-19 pandemic on national immunization programs [7]. In 2022 alone, approximately 17,865 rubella cases were reported across 78 countries, predominantly in Africa and Southeast Asia—regions with the highest CRS incidence. In this context, the Immunization Agenda 2030 and the PAHO Regional Immunization Plan 2023–2030 aim to consolidate progress and ensure that no one is left behind. Vaccination thus transcends individual protection and constitutes an indispensable public health strategy for sustaining elimination, preventing viral reintroduction, and safeguarding pregnant women and newborns from the devastating consequences of CRS [3,6,7].

## 2. Method

We conducted a systematic search in the main international scientific databases, including PubMed/Medline, SCOPUS, and the regional database SciELO. The search strategy was designed to identify studies addressing rubella and its complications, encompassing a wide range of clinical, epidemiological findings, and postnatal follow-up of infants infected with rubella.

We included full-text articles, regardless of the publication language, to ensure maximum comprehensiveness and minimize language-related bias. Furthermore, we limited the search to studies published within the last 15 years, in order to guarantee the relevance and timeliness of the selected scientific evidence.

The search terms used were related to “Rubella”, “Congenital Rubella Syndrome”, “Rubella Complications”, and their equivalents in other languages when applicable, applied individually and combined with Boolean operators (“AND”, “OR”).

## 3. Etiology

The rubella virus is a single-stranded, positive-sense, enveloped RNA virus that belongs to the *Togaviridae* family, which comprises two genera: Alphavirus, primarily composed of arboviruses, and *Rubivirus*, whose sole species—*Rubella virus*—is the etiological agent of rubella. The virus contains three main structural polypeptides: E1, E2, and C, along with three surface glycoproteins, notably E2 and E1-RV, the latter of which is responsible for the virus’s neurotropic behavior, a key feature of its pathogenicity [8].

Transmission occurs primarily through respiratory droplets or vertically via transplacental passage during pregnancy, with the virus exhibiting marked teratogenic potential. In summary, the rubella virus belongs to a group of viruses that result in a high level of viremia, which is associated with its transplacental transmission.

The incubation period ranges from 14 to 21 days, and the highest transmissibility is observed from seven days before to seven days after the appearance of the characteristic exanthem.

Vertical transmission rates are estimated to reach up to 90% during the first 12 weeks of gestation. This rate decreases between the 12th and 28th weeks but increases again during the final trimester, with maternal–fetal transmission reaching nearly 100% by the end of pregnancy [9].

## 4. Epidemiology

Given the significant pathogenic potential of maternal rubella infection and the resulting CRS in terms of global public health, worldwide immunization and prevention efforts have yielded promising outcomes. According to Grant and Zimmerman, the substantial global reduction in CRS observed between 1996 and 2019 reflects the widespread introduction and expansion of rubella vaccination within national immunization programs, alongside international partnerships aimed at supporting implementation and raising awareness of its importance [10].

Between 2012 and 2022, the number of countries incorporating rubella, mumps, and varicella vaccines into national immunization schedules increased from 132 (68%) to 175 (90%) of the 194 WHO Member States. During the same period, global vaccine coverage among infants rose from 40% to 68%, and reported rubella cases declined by 81%, dropping from 93,816 cases in 2012 to 17,407 in 2022. Additionally, by 2022, rubella elimination had been verified in 98 countries (51%), representing an increase from 84 countries (43%) in 2019 [11].

In Brazil, rubella has been classified as a notifiable disease since 1996, with epidemiological surveillance reinforced from 1999 onward through integration with measles surveillance efforts. As a result of successful interventions such as targeted vaccination campaigns and outbreak response immunization, particularly among women of reproductive age, Brazil received a Certificate of Rubella Elimination from the 30. Pan American Health Organization (PAHO) in 2015 [12].

However, the post-elimination scenario does not mean the absence of risk. The drop in vaccination coverage during the COVID-19 pandemic has highlighted the fragility of maintaining herd immunity, resulting in the resurgence of diseases such as measles. In addition, globalization and the intense movement of people maintain the constant risk of reintroduction of the virus through imported cases, especially in regions with pockets of low vaccination coverage. Faced with these challenges, Brazil has resumed its immunization and surveillance efforts, which culminated, in 2024, in the regaining of the elimination certificate—now covering measles, rubella and congenital rubella syndrome—by Pan American Health Organization. This recognition reinforces, however, the ongoing need for high levels of vaccination coverage and continuous epidemiological surveillance to prevent the reintroduction and spread of these diseases [13].

According to World Health Organization (WHO) estimates from 2024, approximately 100,000 babies are born with CRS each year worldwide, mainly in regions with low vaccination coverage, such as Southeast Asia and Africa. In contrast, previous studies indicate that in 2019, the estimated number of births with CRS was 32,000 (confidence interval 13,000 to 60,000), representing a reduction of around 73% compared to 1996 [3]. In countries that have achieved rubella elimination, such as those in the Americas and Europe, the incidence of CRS remains low. However, the risk of reintroduction of the virus persists due to imported cases and depends on the maintenance of collective immunity [14].

Recent advances in immunization strategies highlight the potential benefits of alternative vaccine delivery methods. Although the current rubella vaccine is administered parenterally and effectively stimulates a Th2 immune response—leading to the production of IgG and IgM antibodies, essential for neutralizing the virus in the bloodstream—recent studies with other viral vaccines, such as influenza and SARS-CoV-2, have demonstrated that intranasal administration can stimulate a Th1-mediated immune response. This includes enhanced mucosal immunity through IgA production and T-cell memory, which may improve the prevention of viral transmission. While intranasal vaccines for rubella are not yet available, these findings suggest a promising direction for future vaccine development. Furthermore, intranasal delivery could improve vaccine acceptability by eliminating the need for injections [15].

## 5. Pathophysiology

Maternal infection with the rubella virus, particularly during the first trimester of pregnancy, can have devastating consequences for the developing embryo [16]. Following primary infection, the virus disseminates through the maternal bloodstream, reaching the placenta and subsequently entering the fetal circulation. Placental infection represents a critical step, as it enables viral access to the fetus even at relatively low maternal viral loads [17].

Once within the fetal organism, the rubella virus demonstrates a marked tropism for rapidly dividing cells, affecting vital tissues such as the neuroectoderm, vascular endothelium, and precursors of cardiac and ocular cells [18]. Direct viral infection of fetal cells induces apoptosis, disrupts cell cycle regulation, and inhibits mitosis, leading to organ hypoplasia and impaired morphogenesis [19]. In addition, the virus interferes with angiogenesis and induces widespread endothelial damage, resulting in vascular lesions that further compromise tissue development [20].

The clinical manifestations of CRS are direct reflections of these underlying pathophysiological mechanisms:

- Sensorineural hearing loss: Results from the destruction of cochlear hair cells and the organ of Corti, along with vascular damage to inner ear structures. Inflammation and secondary hypoxia contribute to irreversible bilateral hearing loss, the most common sequela of CRS [21].
- Congenital cataracts: Caused by direct infection of the lens cells during development. The rubella virus disrupts the normal differentiation of lens fibers, leading to early-onset bilateral opacification [22].
- Congenital heart defects: Especially patent ductus arteriosus and pulmonary artery stenosis, arise from infection of endothelial and mesenchymal cells during cardiac embryogenesis. Vascular injury and impaired remodeling of cardiac structures result in abnormal formation of the great vessels [23].
- “Salt and pepper” retinopathy: Derives from retinal cell destruction and pigmentary changes secondary to viral infection and vascular damage [24].
- Microcephaly and neurodevelopmental delay: Occur due to infection of neuronal progenitor cells, triggering neuronal apoptosis and disrupting cortical cell migration [25]. Moreover, persistent inflammation in the central nervous system contributes to progressive brain injury.

The risk and severity of these sequelae are closely linked to the gestational age at the time of infection. Infections during the first trimester have a vertical transmission rate exceeding 80% and are associated with the highest risk of severe anomalies, which may result in miscarriage or stillbirth. In contrast, infections acquired after 20 weeks of gestation carry a significantly lower risk of structural malformations [26]. Persistent fetal viral infection is also associated with chronic low-grade inflammation, which contributes to delayed injury of the central nervous system and sensory organs [27].

Thus, the pathophysiology of intrauterine rubella infection involves a combination of direct cellular injury, vascular disruption, and sustained inflammatory responses, culminating in a broad spectrum of congenital anomalies—most of which are preventable through adequate immunization of the population [27].

## 6. Clinical Manifestations

CRS results from maternal rubella virus infection during pregnancy, particularly during the first trimester, a period of heightened fetal vulnerability. Vertical transmission of the virus can lead to a wide range of congenital anomalies [28].

The classic presentation includes the pathognomonic triad of congenital cataracts, congenital heart disease (commonly patent ductus arteriosus or pulmonary artery stenosis), and sensorineural hearing loss—the latter being the most frequent and permanent clinical manifestation. In addition to these, central nervous system involvement may occur, including neurodevelopmental delay and behavioral disorders [26].

The severity and extent of the clinical presentation are closely related to the gestational age at the time of infection, with the most severe outcomes occurring when infection takes place during the first eight weeks of gestation [29]. Table 1 shows the clinical manifestation of rubella virus infection and CRS.

## 7. Diagnosis

Investigation of CRS plays a critical role in understanding its pathogenesis and in improving diagnostic, therapeutic, and preventive strategies. Early detection, combined with appropriate intervention, significantly contributes to reducing complications and improving clinical outcomes in affected individuals [32].

Diagnosis can be established through maternal screening during prenatal care, fetal and intrauterine assessment during gestation, postnatal evaluation, and complementary testing [32].

## 8. Maternal Screening

Maternal screening is based on serological testing for specific IgG and IgM antibodies. Currently, Brazil’s Ministry of Health does not recommend routine rubella serological screening during prenatal care. Testing should be reserved for cases where the pregnant woman is symptomatic or has had known exposure to individuals with exanthematous illnesses. If the pregnant woman is asymptomatic, has no prior history of contact, and lacks vaccination records, IgG serology should be performed. A negative result indicates the need for postpartum vaccination, whereas a positive result confirms immunity. In such cases, an IgG avidity test may aid in diagnosis by distinguishing recent infections (low avidity) from past infections (high avidity) [33].

On the other hand, other guidelines, such as those from the US (Centers for Disease Control and Prevention), recommend serological testing for IgG antibodies against rubella before, during, and after pregnancy. Similarly, countries such as the United Kingdom and Canada consider it relevant to screen for immunity to rubella at least during the first prenatal visit in the first trimester. Thus, international guidelines highlight the importance of prenatal testing as an essential preventive measure to protect the fetus against CRS [34].

In Australia, rubella antibody screening is recommended for all women who are planning to become pregnant or who have recently become pregnant, regardless of previous serological results. In addition, seronegative women should still receive the rubella vaccine after giving birth and before being discharged from the hospital [35].

## 9. Fetal and Intrauterine Assessment

Fetal infection can be diagnosed by detecting viral RNA or performing PCR on amniotic fluid collected via amniocentesis, or by identifying fetal IgM in cord blood obtained through cordocentesis. Viral RNA detection in fetal blood is also possible and is considered the most reliable diagnostic method before 20 weeks of gestation [36,37].

**Figure 1:**
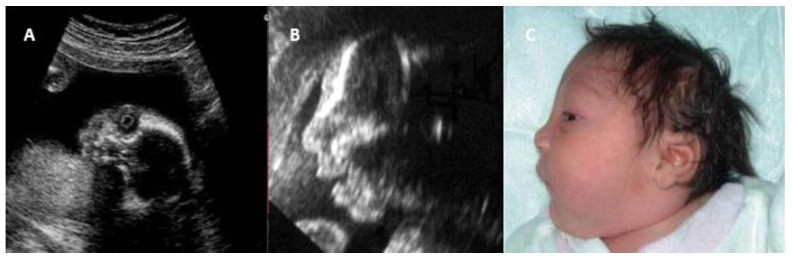
(**A**) Two-dimensional coronal view showing the congenital cataract; (**B**) Two-dimensional sagittal view showing the microcephaly; (**C**) Postnatal imaging confirming the microcephaly.

Ultrasound is another essential diagnostic tool during prenatal care, allowing identification of structural anomalies associated with CRS, such as septal heart defects, pulmonary artery stenosis, microcephaly, cataracts, microphthalmia, and hepatosplenomegaly [38]. Table 2 shows the main intrauterine imaging diagnostic methods suggestive of CRS. Figure 1 shows the ultrasound intrauterine findings and the respective postnatal image of a newborn with CRS.

## 10. Postnatal Evaluation

Detection of rubella-specific IgM antibodies is one of the most commonly employed methods for postnatal diagnosis. However, antibody levels vary with age, as studies have shown IgM to be detectable up to the ninth month of life, and rarely beyond the first year [40]. Another relevant method involves the monitoring of stable or rising levels of rubella-specific IgG antibodies during the first year of life. Since maternally derived IgG is passively transferred via the placenta and typically declines progressively after birth, a persistent or increasing IgG concentration suggests active infection rather than passive immunity [40].

In cases where IgM testing yields a negative result but clinical suspicion of CRS remains, infection cannot be ruled out. In such scenarios, confirmation may be achieved through nucleic acid amplification tests (NAATs), including polymerase chain reaction (PCR), using nasopharyngeal swabs, urine, or oral fluids [41].

Additionally, a thorough clinical evaluation can help detect abnormalities indicative of CRS, such as sensorineural hearing loss, congenital cataracts, and heart defects. In this context, a novel diagnostic approach involving the phylogenetic analysis of the rubella virus isolated from the lens tissue has emerged as a promising method, particularly for patients presenting with ocular findings [42].

## 11. Differential Diagnosis

CRS may present with varying degrees of severity and, unlike many other congenital infections, has a strong correlation with the timing of maternal infection. Exposure during the first trimester is particularly associated with severe outcomes. Importantly, rubella remains the only congenital infection that can be effectively prevented through vaccination, which has substantially contributed to the decline in its global incidence [11,30,43].

For this reason, early and accurate diagnosis is crucial, as is the ability to distinguish CRS from other congenital infections. However, this task can be challenging due to the overlapping clinical features among these conditions [44].

In this context, certain findings—such as specific congenital heart defects and distinctive ocular abnormalities like the characteristic “salt and pepper” retinopathy—are particularly helpful for the early and accurate identification of CRS. These features support timely prenatal interventions and effective postnatal management [45]. Table 3 shows the main prenatal differential diagnosis of CRS.

## 12. Postnatal Findings

Prospective surveillance studies of infants presenting with clinical features suggestive of CRS have identified several key postnatal findings. The most frequently reported outcomes include low birth weight (71%), congenital heart defects (72%), cataracts (13%), hearing impairment (93%), purpura (84%), hepatosplenomegaly (68%), and thrombocytopenia (76%). Neonatal death is also notably frequent. A striking finding reported in 84% of these cases was the presence of purpuric skin eruptions, commonly described as the “blueberry muffin baby” appearance [47].

Among cardiovascular complications, patent ductus arteriosus was the most common, often associated with the development of progressive pulmonary hypertension (PH). Mortality in infants with CRS was frequently attributed to PH, especially around six months of age. However, in many cases, surgical closure of the ductus arteriosus led to reversal of this condition [47].

Ophthalmologic examinations revealed characteristic features of central retinal chorioretinopathy on fundoscopy [48]. A distinctive “salt and pepper” retinal appearance—resulting from alternating hypo- and hyperpigmented areas—was frequently observed [49]. Fluorescein angiography often demonstrated irregularly distributed spots of hypoautofluorescence [2], which may reflect either abnormal melanin production or melanin loss in the retinal pigment epithelium, a key site of rubella-induced ocular infection. Additional ocular abnormalities observed in these patients include cataracts, myopia, hyperopia, strabismus, microphthalmia, and nystagmus, which may result in mild or asymptomatic visual impairment in many affected individuals [49].

Regarding hearing function, most children exhibited moderate to profound bilateral hearing loss. Other nonspecific otologic findings included unilateral or bilateral otitis media, often with tympanic membrane abnormalities [50].

In a study incorporating photographic documentation, facial dysmorphisms were identified in 54% of children with CRS. The most common findings were a broad forehead, followed by a low anterior hairline with whorl. Other features included microcephaly, triangular face, prominent nose, and micrognathia. Although ocular abnormalities were not significantly associated with facial dysmorphisms, congenital heart defects were strongly correlated with these features [51].

Longitudinal follow-up of children who survived congenital rubella infection revealed global developmental delays. In a two-year follow-up study, 95% of children were suspected of having developmental difficulties, failing at least one domain of the Ages and Stages Questionnaire (ASQ), with the most affected areas being communication and language, as also reflected in the Denver II test. Furthermore, 41% of the children were suspected to be on the autism spectrum, with predominant deficits in problem-solving and social-personal domains [52].

These findings underscore that rubella infection carries high mortality, and surviving children may experience a wide range of lifelong disabilities of varying severity and combinations, which may emerge or worsen during adolescence or adulthood. Therefore, achieving high vaccine coverage is crucial to the prevention of CRS, while early detection and comprehensive management are vital to improving quality of life throughout the lifespan [52]. Table 4 shows the main signs and symptoms of infants with CRS. 

## 13. Prognosis

The prognosis of Congenital Rubella Syndrome (CRS) is complex and depends on the severity of clinical manifestations at birth, the extent of organ damage, and access to specialized medical care [53]. Surveillance studies show that most affected newborns present with multiple anomalies, including congenital heart defects—such as patent ductus arteriosus and pulmonary artery stenosis—congenital cataracts, pigmentary retinopathy, microcephaly, and sensorineural hearing loss, the latter being among the most frequent and persistent sequelae [17]. Pulmonary complications, particularly progressive pulmonary hypertension, significantly contribute to early mortality, especially when surgical closure of the ductus arteriosus is not achieved [22].

Beyond these evident physical manifestations, survivors of CRS often experience global neurodevelopmental delays, including deficits in communication and language, behavioral abnormalities consistent with autism spectrum disorder, and variable cognitive impairment [16]. Longitudinal studies have shown that over 90% of children followed for two years exhibited developmental delays as assessed by tools such as the Ages and Stages Questionnaire (ASQ) and the Denver II test [54]. These difficulties tend not only to persist but may worsen with age, requiring intensive rehabilitation, specialized education, and long-term multidisciplinary support [54].

Ophthalmological sequelae—including salt-and-pepper retinopathy, cataracts, and microphthalmia—also have a lasting impact on quality of life and often necessitate early surgical intervention and lifelong ophthalmologic follow-up [55]. Cardiac anomalies, if not corrected in a timely manner, may progress to severe heart failure and early mortality [56]. Overall, the prognosis of CRS involves high neonatal mortality, a high prevalence of multiple sequelae, and a lifelong need for continuous medical care and support.

## 14. Short- and Long-Term Follow-Up

Deficits associated with Congenital Rubella Syndrome (CRS) vary across the lifespan, influencing behavioral development and functioning in both educational and domestic environments [57]. Many impairments are intrinsic to CRS, with sensory deficits—especially hearing loss and visual impairment—being most prominent. Early childhood is frequently marked by abnormal muscle tone and reflexes, motor delays, feeding difficulties, and altered clinical behavior. Global development is substantially delayed; for instance, affected children have been reported to achieve head control only by 14 months (expected at 3 months) and fail to sit independently by 22 months (normally achieved by 7–8 months) [50].

During later childhood, worsening of hearing loss, motor impairments, balance disturbances, and behavioral disorders are observed. In adolescence and adulthood, long-term sequelae include learning disabilities, behavioral disturbances, deteriorating motor coordination, muscular weakness, and impaired tactile perception [57].

Developmental delays have been assessed using tools such as the ASQ and Denver test, with significant deficits reported in communication (82%) and language (76%). Persistent difficulties were noted in communication, language, and psychosocial problem-solving, especially among children with hearing impairment [50].

In addition, children exhibiting developmental delay were screened for autism risk using the Modified Checklist for Autism in Toddlers (M-CHAT), with 41% identified as suspected cases of the condition. Subsequent evaluation according to DSM-IV criteria confirmed diagnoses of autism spectrum disorder in 3 of the 17 children evaluated, with greater severity observed in females, all of whom also had hearing and visual impairments [50].

Early intervention—including occupational, speech, and physical therapies—is fundamental to improving quality of life and reducing morbidity and mortality. Ongoing, integrated care by a multidisciplinary team is essential to tailor interventions to the complex needs that may evolve throughout the child’s development [52].

## 15. Conclusions

Intrauterine rubella infection remains a serious threat to maternal and child health, particularly in populations with insufficient vaccination coverage. Despite global progress toward rubella elimination—especially in the Americas—the risk of disease reemergence persists due to gaps in immunization programs, vaccine hesitancy, and the recent disruptions in healthcare systems caused by the COVID-19 pandemic [58].

Congenital rubella, characterized by multiple structural anomalies and severe neurodevelopmental impairments, is a leading cause of preventable childhood morbidity and mortality [17]. Given the absence of specific antiviral therapy, active immunization remains the only effective strategy for preventing primary rubella infection during pregnancy and, consequently, the development of CRS [56].

Vaccination campaigns, coupled with integrated epidemiological surveillance and public health education, are essential to sustaining elimination efforts and preventing viral reintroduction [58]. Furthermore, early identification of suspected CRS cases and timely implementation of diagnostic and therapeutic measures are crucial for minimizing long-term sequelae [59].

Children affected by CRS require multidisciplinary follow-up involving pediatrics, cardiology, ophthalmology, audiology, neurology, and rehabilitation services to optimize developmental outcomes and enhance quality of life [60]. Therefore, CRS represents not only an individual medical condition but also a pressing public health challenge that demands continuous and coordinated efforts to maintain rubella elimination and protect future generations from the devastating consequences of this vaccine-preventable infection [58].

## Figures and Tables

**Table 1:** Clinical manifestations of rubella virus infection and Congenital Rubella Syndrome (CRS).

Clinical Condition	Clinical Manifestation
Acute rubella infection (symptomatic)	- Low-grade fever
	- Erythematous maculopapular rash with cephalocaudal progression
	- Lymphadenopathy (retroauricular, cervical, occipital)
	- Arthralgia and arthritis, especially in adult women - Purpuric rash (blueberry muffin rash)
	- Idiopathic thrombocytopenic purpura (rare) [28,29,30]
Acute rubella infection (asymptomatic)	- Absence of clinical signs in approximately 25–50% of cases [26]
Congenital Rubella Syndrome (CRS)	- Congenital cataracts
	- Sensorineural hearing loss
	- Congenital heart defects (e.g., patent ductus arteriosus, pulmonary artery stenosis)
	- Pigmentary retinopathy
	- Neurodevelopmental delay
	- Behavioral disorders
	- Endocrine dysfunctions (e.g., congenital hypothyroidism, type 1 diabetes mellitus) [26,28,30,31]

**Table 2:** Intrauterine findings suggestive of Congenital Rubella Syndrome (CRS) [38,39].

Intrauterine Findings	Description	Imaging Methods
Intrauterine growth restriction	Fetal size below expectations for gestational age	US
Microcephaly	Fetal head circumference below normal	US, MRI
Ventriculomegaly/calcifications	Cerebral ventricle dilation or intracranial calcifications	US, MRI
Hydrops fetalis	Fluid accumulation in fetal compartments and subcutaneous tissue	US
Congenital heart disease	ASD, VSD, PDA	Doppler US, fetal cardiac MRI
Hepatosplenomegaly	Enlarged liver and spleen	US
Pleural effusion/ascites	Fluid accumulation in pleural or abdominal cavities	US
Cataract and microphthalmia	Lens opacity and small eye dimensions	Ocular US, MRI
Liver hypoechogenicity	Low echogenic areas suggesting inflammation or infection	US
Pulmonary artery stenosis	Narrowing of the pulmonary artery	Doppler US, fetal cardiac MRI
Echogenic bowel	Increased bowel echogenicity, similar to bone	US
Polyhydramnios	Excess amniotic fluid	US
Placentomegaly	Thickened placenta for gestational age	US

ASD: atrial septal defect; MRI: magnetic resonance imaging; PDA: patent ductus arteriosus; US: ultrasound; VSD: ventricular septal defect.

**Table 3:** Main prenatal differential diagnoses of rubella and other intrauterine infections, and the presence of vaccination.

Characteristic	Zika Virus	Rubella	Cytomegalovirus (CMV)	Toxoplasmosis
Gestational age period with higher risk	First trimester (7–13 weeks) [45]	First trimester (particularly around the 10th week) [45]	First trimester, with risk persisting across all trimesters [45]	Third trimester (predominantly during the ninth month) [44]
Adverse perinatal outcomes	Microcephaly (also seen in rubella and CMV), ocular anomalies [46]	Sensorineural hearing loss (as in CMV), cataracts, cardiac anomalies, microcephaly [45]	Sensorineural hearing loss, chorioretinitis (as in toxoplasmosis), microcephaly, cerebral palsy, intellectual disability [45]	Intracranial calcifications (50–80%), hydrocephalus, and chorioretinitis [44]
Brain calcification pattern	Predominantly subcortical [44]	Periventricular and basal ganglia [44]	Periventricular (similar to rubella) [44]	Not typically described with a specific calcification pattern
Vaccination availability	No	Yes [11]	No	No

**Table 4:** Postnatal clinical findings in infants with Congenital Rubella Syndrome (CRS).

System	Signs and Symptoms
Vision	- Central serous chorioretinopathy
	- Congenital cataract
	- Salt-and-pepper retinal pigmentary changes
	- Microphthalmia
	- Glaucoma
	- Irregularly distributed speckled hypoautofluorescence
	- Macular neovascularization
	- Myopia
	- Hyperopia
	- Strabismus
	- Nystagmus
Hearing	- Sensorineural hearing loss
Facial Dysmorphisms	- Triangular face
	- Microcephaly
	- Broad forehead
	- Low anterior hairline
	- Whorl on anterior hairline
	- Prominent nose
	- Micrognathia
Cardiac	- Patent ductus arteriosus (PDA)
	- Pulmonary artery stenosis
	- Atrial septal defect (ASD)
	- Ventricular septal defect (VSD)
	- Cardiomyopathy
	- Arrhythmias
Pulmonary	- Pulmonary hypertension
Neurodevelopment	- Communication difficulties
	- Language delay
	- Autism spectrum disorder

## Data Availability

The data presented in this study are available on request from the corresponding author.
